# A journal that grows older and stays young

**DOI:** 10.1128/jb.00340-26

**Published:** 2026-08-14

**Authors:** Tao Dong

**Affiliations:** 1 Immunology and Microbiology, School of Life Sciences, Guangming Advanced Research Institute, Southern University of Science and Technology 255310, Shenzhen, Guangdong, China; Dartmouth College Geisel School of Medicine, Hanover, New Hampshire, USA

**Keywords:** history, China, education, bacteriology

## EDITORIAL

Few journals in microbiology carry the history, continuity, and influence of the *Journal of Bacteriology* (JB). Since its founding in 1916, JB has published for more than a century and has served as a venue for discoveries that shaped how we understand bacteria and their physiology, genetics, metabolism, evolution, interactions, and remarkable adaptability (1). For many of us bacteriologists, JB is not only a journal we read, submit to, review for, or edit. It is also part of our personal scientific story.

I still remember a conversation near the end of my postdoctoral training at Harvard Medical School. During a usual Friday Beer Hour, a young professor in the department told a group of us that for a new principal investigator, it was important to establish independence quickly. Publishing a strong paper in JB, he suggested, was an excellent way of showing that. Now, looking back as someone more senior, as a frequent grant and paper reviewer, and as an editor, I have gained more appreciation for that practical career advice. Over the 54,000 papers JB has published to date, many surely helped young investigators define their scientific identity, demonstrate independence, and build their careers.

There is little doubt that JB has long been an authoritative voice in bacteriology worldwide. But influence is not only measured by discoveries and citations but also carried through the ways many students first encounter the field. When I was an undergraduate student at Shandong University, access to scientific literature was very different from today. To find the papers I needed, I went to the biology library, searched through printed literature indexes, and photocopied articles from hardbound issues of JB. Learning how to do this was itself part of my training, as a literature-search course was included in the curriculum at that time. Long before I became an author or editor, hardbound issues of JB sitting on the shelves of a university library on the other side of the globe (i.e. in Shandong in my case, the home province of Confucius) were among the sources of scientific education for a young student learning bacteriology.

From the vast and historical literature published by JB, one can learn not only scientific knowledge but also legacies of pioneers. Out of curiosity, I conducted a quick search for authors based in China who had published in JB and found that such publications appeared much earlier than I had expected. Some of the early Chinese authors were trained in both China and the United States. Their careers remind us that international interaction between the two countries in microbiology can be traced to the very early history of the field itself, and that JB was one of the journals in which that history was recorded.

One figure who immediately stood out was Dr. Fei-Fan Tang (汤飞凡), one of the pioneers of modern microbiology in China. After medical training in China, Tang studied at Harvard under Hans Zinsser and published early work on herpes virus, vaccinia virus, ultrafiltration, adsorption, and viral cultivation. He later returned to China, where he became a central figure in epidemic prevention, vaccine and biological-product development, and infectious disease research. In 1932, Tang published what appears to be one of the earliest JB papers with an author address in China. His single-author article, “Adsorption Experiments with the Virus of Vaccinia,” was published from the Department of Bacteriology, Medical College, National Central University, Woosung, Shanghai, China (2). At a time when the physical nature of viruses was still being defined experimentally, studies of filtration, centrifugation, cultivation, and adsorption were essential for understanding infectious agents that passed through filters and could not yet be readily visualized. His most famous achievement was the isolation and cultivation of the trachoma agent, later recognized as *Chlamydia trachomatis*, a landmark discovery in medical microbiology (3, 4). His early presence in JB is therefore more than a historical curiosity to me; it highlights a scientific career shaped by international training, experimental rigor, and the building of microbiology in China.

Another example is Shu-Hsien Wang, or Wang Shuxian (王叔咸), who published in JB in 1941 from the Department of Medicine, Peiping Union Medical College, Peiping, China. Wang was trained at Peking Union Medical College, an institution itself deeply shaped by international medical education, and later became one of the major figures in modern Chinese internal medicine. Early in his career, he worked on tuberculosis, diabetes, and metabolic disease; later, he became a founding figure of nephrology in China. His 1941 JB paper, “A Direct Smear Method for Counting Microscopic Particles in Fluid Suspension,” was a methodological study (5). It may appear modest at first glance, but it reflects an important tradition in bacteriology: careful measurement, reproducible technique, and quantitative thinking. Such methodological papers are part of the backbone of the field, making organisms countable and experiments reproducible across laboratories.

A third early example is H. Zanyin Gaw, or Gao Shangyin (高尚荫), who published in JB in 1944 from the Department of Biology at National Wuhan University. His paper, “Isolation and Study of Cultures of Chinese Vetch Nodule Bacteria,” examined symbiotic nitrogen-fixing bacteria associated with Chinese vetch (6). Gaw had studied biology in China and abroad, including at Rollins College and Yale, before returning to China and helping develop microbiological research and education. His work reminds us that early bacteriology in China was also broad in scope, extending beyond medical microbiology to agricultural and environmental microbiology.

These early papers are remarkable because they appeared during a prolonged period of brutal war and mass population displacement, a profoundly difficult time in modern Chinese history. This history matters. It shows that JB had international readers and authors from very early on, even including scientists working under exceptionally difficult conditions. It also reminds us that scientific journals are more than archives of papers. They are communities across time. A paper published in JB may serve many roles: a technical reference, a conceptual advance, a young investigator’s first independent statement, or a bridge connecting bacteriologists across countries and generations.

As an editor (and the first editor for JB based in China), I find this deeply meaningful. JB now has a 110-year history, but its mission remains current: to publish rigorous, mechanistic, and important work in bacteriology. The field continues to change, and so does the journal. New technologies, new organisms, new ecological contexts, and new generations of scientists continually renew JB’s scientific life. A journal accumulates wisdom with age, but it stays young when it continues to welcome new questions, new authors, and new communities of scientists. Its strength lies not only in its history, but also in its capacity for renewal. Like the bacterial systems we study, JB persists, adapts, and regenerates: older in wisdom, yet young in spirit.
